# Epidemiology of Swine Brucellosis in Selected Districts of East Shewa Zone, Ethiopia

**DOI:** 10.1155/vmi/9938144

**Published:** 2025-09-24

**Authors:** Aregawi Girmay Gebreegziabher, Gezahegne Mamo, Fufa Abunna

**Affiliations:** College of Veterinary Medicine and Agriculture, Addis Ababa University, P.O. Box 34, Bishoftu, Ethiopia

**Keywords:** Alage, Batu, brucellosis, Naka, pig, seroprevalence

## Abstract

Our world has been threatened by numerous emerging and reemerging pathogenic diseases; these diseases are seriously affecting the well-being of human, animal health, and animal production. Most of them are zoonotic diseases and have great veterinary and public health impact, particularly in developing countries where people are having daily frequent contact with livestock and animal products. Brucellosis is one of them. A cross-sectional study aimed to determine the seroprevalence and associated risk factors of swine brucellosis was carried out at Batu, Alage, and Naka village using serological tests. Accordingly, a total of 196 pigs were included in the study. Rose Bengal Plate Test (RBPT) prepared from the smooth strain *B. abortus* antigen was used as a screening test, whereas Complement Fixation Test (CFT) was the confirmatory test for swine brucellosis. The results of the present study indicated that, of the total 196 serum samples from pigs, 10 (5.1%) were found to be positive by RBPT and 7 (3.6%) of them were further confirmed by CFT. On the other hand, age and history of obstetrical problems were the major risk factors for overall swine brucellosis seropositivity (*p* < 0.05). Thus, the present study suggests that swine brucellosis is prevalent in the study areas and the seropositivity could give an insight that brucellosis could pose public health hazard. Hence, this warrants public education and awareness, and further extensive epidemiological and molecular investigation is recommended.

## 1. Introduction

Brucellosis is an ancient and one of the world's most widespread zoonotic diseases accounting for the annual occurrence of more than 500,000 human cases, affecting both public health and animal production [1]. It is primarily a disease of the reproductive tract of animals mainly characterized by late stage of abortion, infertility, retention of placenta, reduced milk yield, orchitis, and epididymitis in animals and undulant fever, headache, sweating, and other complications in humans [2].

It is endemic in the Middle East, Mediterranean countries, Asia, Africa, and Central and South America and consistently ranked among the most economically important zoonoses globally, as it results in reduced productivity, abortions, weak offspring, and major impediments for trade and export of livestock. It poses a barrier to trade of animals and animal products between countries and causes considerable economic losses which can lead to international trade ban [3, 4].

In many high-income countries, it has been successfully controlled both in livestock populations and humans. However, in low-income countries like Ethiopia, it is endemic with large disease and livelihood burdens in animals and people and very weak effective control [5]. Since the first report of brucellosis in the 1970s in Ethiopia, the disease has been noted as one of the important livestock diseases and has been reported from different localities, commonly targeted on bovine, occasionally sheep and goats, and rarely camels [6]. It has been reported that infected dogs have a role in spreading of *Brucella abortus, Brucella suis*, and *Brucella melitensis* to neighboring herds, flocks, and humans [7, 8].

In Uganda, a 2016-17 cross-sectional survey found < 1% prevalence (none positive by confirmatory test or PCR); brucellosis risk from pigs appears *negligible* in pigs under typical production systems [9]. In Kenya, a 2020 study detected 0.57% prevalence by RBT, with some PCR-positive but cELISA-negative samples, and identified *Brucella abortus* DNA in some pig sera—indicating pigs may harbor *Brucella* spp. but rarely transmit it [10]. A 2022 review highlights seroprevalence studies in Central Ethiopia (2015–2022), with pig-level values of 3.8%–5.1% by RBT or iELISA, but these are few and outdated and often lack confirmatory testing [11]. Pig farming in Ethiopia is still small overall, but it is booming in certain pockets because of a mix of economic, dietary, and market factors that are starting to outweigh the traditional barriers. Urbanization and the growth of expatriate communities have created a steady demand for pork in few major cities in Ethiopia. Moreover, hotels, restaurants, and supermarkets now cater to these customers with pork dishes and processed pork products. Smallholder and semi-commercial pig farms are mainly found in peri-urban areas of Central Ethiopia including the study area.

Though the population of pigs has shown increment in the central part of the country, the occurrence and epidemiology of brucellosis in these animals are poorly understood. Therefore, this study is designed with the aim to estimate the seroprevalence of brucellosis in swine, evaluate the degree of association of potential risk factors, and assess the awareness and practices of swine farm employees toward the disease.

## 2. Materials and Methods

### 2.1. Description of the Study Area

The study was conducted at Batu, Alage, and Naka village. Batu is located at 163 km from the capital Addis Ababa. Alage is located at 217 km southwest of Addis Ababa and 32 km west of Bulbula town, near the Abijatta and Shala lakes of the Ethiopian Rift Valley. Naka village is located at northeast of Alage in Adami Tullu and Jido Kombolcha district, Oromia regional state. Both the college and Naka are geographically located at a longitude of 38°30′ east and latitude of 7°30′north, with an altitude of 1600 m.a.s.l. The mean annual minimum and maximum temperature ranges from 11°C to 32°C, respectively. The areas have a mean annual rainfall of 800 mm and three distinct seasons: a short rainy season (March to May), a long rainy season (June to September), and a dry season (October to February) [10]. Batu is a town and separate district, located on the road connecting Addis Ababa to Hawassa in the East Shewa Zone of the Oromia Region of Ethiopia. It has a latitude and longitude of 7°56′N 38°43′E with an elevation of 1643 m above sea level.

### 2.2. Study Design

A cross-sectional study was carried out at Alage, Batu, and Naka using serological tests on pig sera, so as to determine the seroprevalence of swine brucellosis, respectively. The districts were conveniently selected based on the presence of swine farms.

### 2.3. Study Population

Pigs older than 6 months were the study populations for this study as the disease is not common in < 6 months due to maternal immunity. None of the animals were also vaccinated against brucellosis.

### 2.4. Sample Size and Sampling Method

The required sample size was determined based on expected prevalence of brucellosis [13].

(1) $$\begin{array}{c} n=\frac{1.96^{2}\text{ Pexp }\left(1-\text{Pexp}\right)}{d^{2}}, \end{array}$$

where *n* = the required sample size; *P* = estimated prevalence = 0.5; *Z* = level of confidence as 1.96; and *d* = desired precision level = 0.05.

As the total number of pigs in the two swine farms was small (*n* = 235), all pigs older than 6 months were sampled. Therefore, a total of 196 pigs were included (167 from Alage swine farm and 29 from Batu swine farm).

#### 2.4.1. Sample Collection

After proper restraining, about 6–8 mL of blood was aseptically collected through the saphenous (femoral) vein and ear vein of each pig. Each sample was coded and transported to Alage Agricultural TVET College Department of Animal Health Microbiology Laboratory. The blood samples were allowed to clot and centrifuged at 3000 rpm for 5 minutes. Serum samples were then decanted, transferred into labeled cryovials, and screened by Rose Bengal Plate Test (RBPT). Pigs' sera tested positive by RBPT were stored at −20°C and sent to NVI (Bishoftu, Ethiopia) and Yulin Animal Disease Prevention and Control Center (Yulin city, China), respectively, for further confirmation.

#### 2.4.2. Serological Tests

Rose Bengal *Brucella* antigens (*B. abortus* and *B. canis*) with their corresponding control sera (sourced from the China Institute of Veterinary Drugs Control), as well as Complement Fixation Test (CFT) *Brucella* antigen, control sera, and complement (Bg vv, Germany, and China Institute of Veterinary Drugs Control), were used for the RBPT and CFT assays, respectively. Samples were considered positive for swine brucellosis caused by smooth strains if they tested positive with CFT.

#### 2.4.3. RBPT

All pig serum samples were screened by RBPT containing *B. abortus* antigen [14]. Using a micropipette, 1 drop (30 μL) of the test serum was placed on one spot of the slide. Using another pipette, an equal volume of RBPT antigen was placed close to the test serum on the slide. Using an applicator stick, the antigen and the test serum were mixed thoroughly; the slide was then hand-rocked for about 4 min after which the slide was examined for agglutination under a good source of light. Formation of pink granules (agglutination) was recorded as positive while absence of pink granules (agglutination) was recorded as negative.

#### 2.4.4. CFT

Pig samples positive for RBPT containing *B. abortus* antigen were further confirmed by CFT. The confirmation of swine sera was undertaken at Yulin Animal Disease Prevention and Control Center, Veterinary Comprehensive Laboratory, Yulin city, China. All the reagents required for CFT were evaluated by titration. Sheep red blood cell (SRBC) suspension was prepared before being used in the test. The preparation of reagents and CFT procedures were performed according to the protocols of the Federal Institute for Consumer Protection and Veterinary Medicine Service Laboratory, Berlin, Germany [15]. The CFT test was regarded as positive when the reading is as complete fixation or partial hemolysis and as negative (0) when there is complete hemolysis [16].

#### 2.4.5. Questionnaire and Interview

The study had clearly explained to the respondents and informed consent was obtained. The questionnaire, originally prepared in English, was translated to the local language, Amharic and Afaan Oromo, and back translated to English by independent persons to ensure consistency, clarity, and sociocultural acceptability in the communities. Validation of the questionnaire was conducted by pretesting on a total of 10 individuals to analyze and validate the degree to which the questions were properly understood or misunderstood, the degree to which individuals within a group interpreted the questions differently, the effectiveness of the questions in soliciting the proper information, and any areas of information which were neglected by the proposed questionnaire. Once analysis has been completed, some questions were modified. Lastly they were interviewed on their demographic factors, awareness toward the disease, and on history of the animals and possible factors associated with occurrence of brucellosis. Questions regarding religion, assisting bitches during whelping, and consumption habit of pork were not included due to being a sensitive topic culturally.

The information for hypothesized explanatory variables was gathered from the dog owners, swine farm managers/owners, farm employees, and farm records. Factors like age, sex, history of obstetrical problems (abortion, retained placenta, abnormal vaginal discharges, and infertility for females and swelling of testicle and scrotum dermatitis for males) were recorded for each animal. Age of pigs was categorized as two (≤ 3 years and > 3 years). Furthermore, living status (condition) was categorized as indoors, outdoors, and semi-indoors (modified from [17, 18]).

### 2.5. Ethics Approval

All procedures were carried out according to the experimental practice and standards approved by the Animal Welfare and Research Ethics Committee at Addis Ababa University, College of Veterinary Medicine and Agriculture (AAU CVMA) in accordance with the international guidelines for animal welfare. Samplings of animals were then held with formal and verbal consent from the animal's owners who had been informed about the purpose of the study.

## 3. Results

Among the 196 serum samples of pigs, 10 (5.1%) tested RBPT positive and 7 (3.6%) of them were further confirmed by CFT. Hence, the overall seroprevalence of swine brucellosis in the study area was 3.6% (Table 1).

Table 2 shows a significant association (*p* < 0.05) with age and history of obstetrical problems. Swine brucellosis was found to be higher in > 3 years old (10.64%) than ≤ 3 years old (1.34%) and in those with history of obstetrical problems (20.0%) than those that had not (2.2%).

The magnitude and association of putative variables with *Brucella* positivity by univariable logistic regression analysis (Table 3) indicated that prevalence of swine brucellosis did not show significant variations among farms and sexes (*p* ≥ 0.05). Other risk factors like age and history of obstetrical problems were however highly significant. The odds of brucellosis in pigs > 3 years old were 8.8 times higher than those ≤ 3 years old. Similarly, pigs that had history of obstetrical problems had 21.58 times higher odds of getting infection with brucellosis than those without such history.

Table 4 shows the simplified multivariable logistic regression association of risk factors of swine brucellosis. Variables that were not statistical significant in univariable analysis were reduced based on the stepwise function procedure. Thereby, a reduced model with only two significant predictors (age and history of obstetrical problems) was created. Pigs > 3 years old had higher infection (OR = 9.4, CI; 1.5, 58.9) compared to those ≤ 3 years old, and pigs with history of obstetrical problems had higher seropositivity (OR = 23.2, CI; 4.0, 136.1) in comparison with those who had no such history.

From the total 13 swine farm employees, majority of them were male (69.2%) and between 30 and 45 years old (46.2%) and had elementary education (38.5%) (Table 5).

As shown in Table 6, 84.62% of swine farm employees did not know the disease; 53.85% of them assisted farrowing; 46.15% of them gave aborted materials to dogs; only 20.82% knew they can get any diseases from contact of aborted sow's fetus; and 69.23% of them did not take any precaution while assisting farrowing, disposing aborted material, and cleaning their house.

## 4. Discussion

The overall prevalence of swine brucellosis observed in the study area was 5.1% using RBPT and 3.57% using CFT. This finding is comparable to a previous report from Ethiopia [9], which documented a 4.5% seroprevalence using RBPT, and aligns with the results of Rahman [19], who reported a 4.8% prevalence (RBPT and SAT) in Bangladesh. It is also almost equal with the study by Sharma [20] who investigated a prevalence of 3.59% from Nepal, using iELISA. However, the prevalence observed in the present study is higher than the reports from the authors in [9, 10, 21, 22] which documented seropositivity rates of less than 1%, including no positives by confirmatory tests or PCR, 0.57% by RBT, some PCR-positive but cELISA-negative samples, and 0% prevalence in pig farms from Uganda, Kenya, Nigeria, and Croatia, respectively. In contrast to this, a higher prevalence was reported from Nepal (13.6%) using SAT [23]. An investigation done in 2008 in Croatia pig farms [22] also revealed a prevalence of 21.6%, 21.1%, and 49.0% using RBPT, CFT, and ELISA, respectively.

The difference in seroprevalence of swine brucellosis in the different countries might because of difference in agro-ecological zone, swine production systems used, total pig population in the countries, pork consumption culture of the countries, management systems used, contact with feral pigs/wild pigs/wild boars, sample size used, sampling method, and capacity of the diagnostic tests used. They are then becoming a risk for transmission of *B. suis* to local domestic livestock, including pigs. The review added that brucellosis in swine has reemerged in different European countries as a result of spillover from the wild boar brucellosis (*B. suis* biovar 2) reservoir, particularly in outdoor reared pigs. Besides, in a study conducted by the authors of [24] in the Iberian Peninsula, high apparent prevalence of brucellosis in domestic pigs was found (33%), and wild boar population was found to be seriously affected by *B. suis* biovar 2 infection which might be due to contact between free ranging Iberian domestic pigs and wild boar. Other scholars [25] had also reported *B. suis* in wild animals which could be a source of infection to freely foraging domestic pigs.

There was no significant difference observed between the farms, and this similarity may be due to the similar production system and breeding management. Both farms were intensive type and natural mating was their means of breeding system. Besides, they are located in similar agro-ecological zone and no contact with other animals is reported. This is in correspondence with the report of Rahman [19] who found no significant difference (*p* ≥ 0.05) among different study districts. However, it was reported that there was a significant association of the prevalence of brucellosis with origins of the pigs [9]. They showed highest prevalence in pigs originated from Adama area followed by Addis Ababa area. However, this may be due to the difference in agro-ecological zone and general management system among the study locations. Adama is located at hot lowland of Rift Valley, whereas Addis Ababa is located at highland with humid climate. Moreover, they had sampled in abattoir originated from both intensive and extensive swine farms.

Sex wise, though there was higher prevalence in females (4.8%) than males (1.4%), it was not significantly associated. Similarly, Rahman [19] reported insignificant but high prevalence of brucellosis (5.6%) in females and 2.9% in male pigs in Bangladesh. Other researchers also observed insignificant but higher prevalence in females (15.1%) than that of males (12.0%) [24]. A significant association between sex and seropositivity was observed, with females showing a higher prevalence (8.2%) than males (1.6%) [9]. In contrast, a study from Nigeria [26] reported a relatively higher prevalence of brucellosis in male pigs than in females.

Age wise, using multivariable logistic regression analysis, the odds of the disease in those > 3 years old pigs were 9.44 (CI = 1.51–58.92) times higher compared to those ≤ 3 years old. This finding is consistent with Rahman [19], who reported a higher prevalence of brucellosis in older animals (8.1%) than in younger ones (0.0%), and with [23], who also observed higher prevalence in adults. However, it contrasts with the results of the authors in [9], who found a higher prevalence in younger pigs (5.9%) than in adults (3.6%). In the present study, the higher prevalence of swine brucellosis among sexually mature pigs may be attributed to longer exposure time with age and the maturation of reproductive organs, which are preferred sites for *Brucella* localization [27]. Adult animals have more opportunities over time to encounter another positive animal. Because transmission can be associated with mating, the probability of exposure likely increases once an animal is of breeding age.

The odds of the disease in those with history of obstetrical problems were 23.2 (CI = 3.96–136.13) times higher compared with those without such history and were statistically significant. This finding fits with a research of Rahman [19] who found that the prevalence of brucellosis was significantly higher in aborted or previously aborted sows than the sows having no record of abortion when the sera samples were tested by RBT and SAT. Other researchers [19, 28] had also reported similar findings. They reported a statistically significant association between a history of retained placenta and higher prevalence of the disease. Accordingly, it can be inferred that brucellosis was a principal cause of the reproductive problems observed in the studied swine farms, as the pathogen tends to localize and multiply in the placenta and fetuses of sows, as well as in the testes, epididymis, seminal vesicles, and bulbo-urethral glands of boars.

The result of questionnaire survey revealed that the knowledge and understanding about brucellosis among the dog owners and swine farm employees was very limited. Only 1.5% of dog owners and 15.4% of swine farm employees knew the disease. This finding is consistent with the results from Temeke Municipality, Dar es Salaam, Tanzania who showed low awareness about the disease among dog owners; and with [29], which showed that pig traders and slaughterhouse workers had limited knowledge of swine brucellosis. According to their study, the majority of pig traders (66.7%) and pig dressers (94.4%) had limited knowledge on the occurrence of the disease. In Ethiopia, veterinary medicine is widely recognized as important only for the cattle population. The attention given toward these animals by veterinarians is very poor and so much misunderstanding prevails with regard to preserving their healthy conditions.

Furthermore, only approximately a quarter of swine farm employees (20.8%) were aware of the risk of transmission of any disease to human from contact of sows' aborted material. However, a good knowledge about the disease transmission had a precautionary effect as they perform better preventive practices to avoid contracting brucellosis. This is supported by [30] who explained that good knowledge about the disease transmission among farmers had a precautionary effect for brucellosis. In a similar way, a case control study done by the authors of [31] in Iran demonstrated that having awareness regarding modes of brucellosis transmission was associated with a reduced risk of human brucellosis infection. This suggests that improving the animal owners' knowledge of the disease and its mode of transmission is likely to reduce their risk of brucellosis transmission from dogs and pigs. It is therefore important to establish an educational campaign in the study areas to enlighten the communities on the disease.

Findings from current study also illustrate that among those reported abortion cases, 38.46% of swine farm employee throw out the aborted material to outside environment. Moreover, 46.15% of farm employees gave aborted fetus to dogs. This lack of awareness on proper disposal of the aborted material can lead to the transmission of the disease to other animals and humans. Many *Brucella* organisms are shed during abortion, contaminating the environment and increasing the risk of others [32]. A considerable proportion of farm employees (69.2%) did not use any protective equipment or take precautions—such as wearing coveralls, hand/rubber gloves, boots, or practicing hand washing/disinfection—while disposing of aborted materials or cleaning pig houses. A similar finding was reported in Nigeria [16], where 84% of respondents neither used protective gear nor washed their hands. This poses a significant risk, particularly for more than 50% of swine farm workers in the present study who assisted during farrowing, since the pathogen can enter the body through intact skin or minor abrasions [33].

## 5. Conclusion and Recommendations

The results of the present study revealed that the prevalence of swine brucellosis was 5.1% by RBPT and 3.6% by CFT. Age and history of obstetrical problems were found to be the major risk factors for overall swine brucellosis seropositivity. Thus, the present study suggests that swine brucellosis is prevalent in the study areas which could give an insight that brucellosis could pose public health hazards. Hence, this warrants public education and awareness, and further in-depth epidemiological and molecular investigation is recommended.

## Figures and Tables

**Table 1:** Prevalence of swine brucellosis.

Farm	*n*	RBPT positive (%)	CFT positive (%)
Alage swine farm	167	9 (5.4)	6 (3.6)
Batu swine farm	29	1 (3.5)	1 (3.5)
Total	**196**	**10 (5.1)**	**7 (3.6)**

*Note:* For confidentiality, the name of the farm found in Batu is not actual name. 196 is the total number of samples, 10 (5.1) is those samples positive for RBPT, and 7 (3.6) is those positive for CFT.

**Table 2:** Association of risk factors with seropositivity by Fisher's exact test.

Variables	*N*	Positive samples by CFT (%)	*p* value
Farm			0.723
Alage swine farm	167	6 (3.6)	
Batu swine farm	29	1 (3.5)	
Sex			0.425
Male	70	1 (1.4)	
Female	126	6 (4.8)	
Age			0.009
≤ 3 years	149	2 (1.3)	
> 3 years	47	5 (10.6)	
History of obstetrical problems			0.001
No	181	4 (2.2)	
Yes	15	3 (20)	
Overall	**196**	**7 (3.6)**	

*Note: *196 is the total number of samples diagnosed and 7 (3.6) is those positive for CFT.

**Table 3:** Univariable logistic regression of associated risk factors of swine brucellosis.

Variables	*p* value	OR (95% CI)
Farms		
Alage swine farm	^∗^	
Batu swine farm	0.969	0.96 (0.1–8.3)
Sex		
Male	0.256	
Female	^∗^	0.29 (0.0–2.5)
Age		
≤ 3 years	^∗^	
> 3 years	0.011	8.75 (1.6–46.7)
History of obstetrical problems		
No	^∗^	
Yes	0.001	21.58 (4.3–108.6)

^∗^Reference.

**Table 4:** Multivariable logistic regression association of risk factors of swine brucellosis.

Variables	*p* value	OR (95% CI)
Farm	^†^	^†^
Sex	^†^	^†^
Age		
≤ 3 years	^∗^	^∗^
> 3 years	0.016	9.44 (1.5–58.9)
History of obstetrical problems		
No	^∗^	^∗^
Yes	0.001	23.22 (4.0–136.1)

^∗^Reference.

^†^Removed variables following model simplification by stepwise function (*p* ≥ 0.05).

**Table 5:** Demographic characteristics of swine farm employees (*n* = 13).

Demographic characteristics of the respondents	Category	*n* (%)
Gender	Male	9 (69.2)
	Female	4 (30.8)

Age	< 30 years	5 (38.5)
	30–45 years	6 (46.2)
	> 45 years	2 (15.4)

Education status	Illiterates	1 (7.7)
	Elementary	5 (38.5)
	High school	3 (23.1)
	Certificate	1 (7.7)
	Diploma	1 (7.7)
	Degree and above	2 (15.4)

Total		13

**Table 6:** Awareness and practices of swine farm employees regarding swine brucellosis.

Variable	Category	*N* (%)
Know the disease	Yes	2 (15.4)
	No	11 (84.6)

Know that they can get any diseases from contact of aborted sow's fetus	Yes	81 (20.8)
	No	203 (52.2)
	Not sure	105 (27.0)

Disposal of aborted material	Burning	1 (7.7)
	Burying	1 (7.7)
	Throwing away	5 (38.5)
	Give to dog	6 (46.2)

Assist farrowing	Yes	7 (53.9)
	No	6 (46.2)

Wear any protective equipment/take any precaution before and after handling a pig, assisting a birth, disposing aborted material, cleaning house	Yes	4 (30.8)
	No	9 (69.2)

## Data Availability

The datasets used and/or analyzed during the current study will be available from the corresponding author upon reasonable request.

## Nomenclature

CFT: Complement Fixation Test
NVI: National Veterinary Institute
OIE: Office International des Epizooties
RBPT: Rose Bengal Plate Test
RSAT: Rapid slide agglutination test
SAT: Serum agglutination test
WHO: World Health Organization
